# Examining Effects of a Multisite Youth Outreach Program: A Meta-Analysis Approach

**DOI:** 10.5195/jyd.2021.1055

**Published:** 2021

**Authors:** Weiling Li, Martha Lindley McDavid, Sandra F. San Miguel, Loran Carleton Parker

**Affiliations:** Evaluation and Learning Research Center, Purdue University; College of Veterinary Medicine, Purdue University; Evaluation and Learning Research Center, Purdue University

**Keywords:** STEM outreach, meta-analysis, multisite evaluation, heterogeneity, program effects

## Abstract

This paper presents the application of a meta-analysis approach to the evaluation of youth-learning data from the nationally distributed This is How We “Role” program. The application of meta-analysis for examining the impact of other multisite youth programs encountering similar data analysis challenges is discussed. At each This is How We “Role” program site, university partners collected data to examine youth-participant learning. Data analysis from these unique sites was challenging as the approach had to accommodate the innate heterogeneity across sites due to differences in implementation, sample size, and learning context. The meta-analysis method revealed details of the underlying variation between sites that could be masked by typical regression approaches, estimated overall program effects, examined subgroups and identified heterogeneity across project sites. The results showed the This is How We “Role” program generally increased learning at each site and as a whole, even though the program effects varied across sites. This example demonstrates the utility of using the meta-analysis approach to similar multi-site youth development programs.

## Introduction

Evaluating the impact of multisite youth development programs can be challenging. Even with a consistent program framework, delivery will vary across sites due to differences in personnel, resources, participants, program size, and community needs. The data analysis approach must accommodate the innate heterogeneity to get an accurate picture of impact at individual sites and across all sites. The unique demands of evaluating programs at different geographical locations are referred to as multisite evaluation (Turpin & Sinacore, 1991).

In multisite evaluation, the number of sites, type of site, and the intervention being studied can all vary (Straw & Herrell, 2002). Three relatively distinct types of multisite evaluations are cluster evaluations, multisite (cross-site) evaluations, and multicenter clinical trials (Straw & Herrell, 2002). Most literature on multisite evaluation has come from writings about place-, group-, and cluster-randomized controlled trials (Coryn et al., 2015). Guidance on how to execute uncontrolled multi-site evaluations is sparse and usually treats sites as homogeneous (Coryn et al., 2015). As many multisite implementations are innately heterogeneous across sites, there is a need to understand and accommodate between-site variations in intervention effects. The purpose of this paper is to use meta-analysis to explore the effects of a multisite youth outreach program and to develop learnings to guide the planning, implementation, and analysis of similar youth outreach program evaluations. Specifically, this paper uses meta-analysis to estimate program impact on youth learning and examine variation in program impact on learning across sites.

Meta-analysis is a statistical method for aggregating data from multiple studies (Coryn et al., 2015). Meta-analysis is a synthesis usually used to summarize and make collective interpretations of findings from a series of research articles (Glass, 1976). It is an effective method to conduct between-study comparisons with data from multiple studies that address similar questions. When compared to the findings from a single, stand-alone study, aggregating data and findings across many studies using data analysis yields a more powerful and precise examination of the research question. Pharmaceutical companies use meta-analysis to summarize different studies to assess the efficacy of a new drug. *What Works Clearinghouse* (WWC), a widely cited and premier repository of evidence within the U.S. Department of Education, uses meta-analysis to synthesize effect sizes within educational intervention reports (What Works Clearinghouse, 2020). The fixed-effects meta-analytic average is used by WWC to determine intervention effectiveness rating (What Works Clearinghouse, 2020). Meta-analysis is also commonly used as a tool to summarize findings of multisite randomized controlled trials and quasi-experimental studies in health fields (Banks et al., 2002).

Meta-analysis was developed as a tool for examining pooled effects of multiple studies in order to increase statistical power and confidence in overall findings, as well as to give a clearer picture of the impact of context on study findings (i.e., between-site differences). This analytical approach can be translated to multisite program evaluation to yield the same benefits such as computation of site-specific effect sizes and exploration of relationships among site characteristics and outcomes (Banks et al., 2002). Thus, meta-analysis applied to multisite youth outreach program evaluation can address many of the limitations of traditional statistical approaches.

Coryn et al. (2015) summarized the advantages of meta-analysis as a method for multi-site evaluations as follows:

- Does not assume homogeneity of either the treatment or the sites
- Can accommodate a wide variety of evaluation designs, including non-experimental, quasi-experimental, and experimental, as well as a wide range of primary measures in various forms (e.g. continuous data, binary data, ordinal data)
- Produces effect estimates, or simple point estimates that generally do not require complex or sophisticated interpretation
- Assesses and communicates details of the underlying variation between and within sites, a limitation of most traditional multisite evaluation methods
- Presents results in the form of forest plots to visualize statistical estimates of between-site variability
- Examines subgroups through moderator or meta-regression techniques to aid in the understanding of within- and between-site differences in effects

However, there is limited literature addressing the application of meta-analysis as a useful method when applied to multi-site youth outreach programs and if combining data across sites will contribute to better understanding of these programs. Combining the findings across many sites increases the number of programs and persons in subgroups and, therefore, provides the statistical power required to investigate program-level and subgroup effects. For example, at a single youth program, sample size and diversity of youth may be limited. Meta-analysis increases sample size and diversity by pooling data across sites, allowing for testing of subgroup effects (e.g., gender, socioeconomic status) with increased power. Such analysis is central to the purpose of many youth outreach programs that aim to support the skill and capabilities of young people from diverse backgrounds. The pooling of data using meta-analysis also creates opportunities to test various alternative hypotheses (e.g., comparing variations across sites, synthesizing evaluation findings from different sites), making them potentially applicable to a variety of youth education intervention findings, versus only being able to compare programs with very similar structures, students, and so on. As youth outreach programs grow to multiple locations or similar programs collaborate for an evaluation study, meta-analysis permits within- and across-site analyses without the rigid, and often unrealistic standards for real-life programs, of other analytic approaches (e.g., adhering to a strict intervention protocol that does not accommodate the unique needs of youth and their community). Instead, meta-analysis provides the flexibility to deliver meaningful and contextualized programming to target youth without compromising evaluation design. In this paper, we provide the scientific and applied rationale for the analytic strategy, the process to perform the analysis, and a case study for the application in a multisite study that evaluated the effectiveness of a nationwide youth outreach program.

### Case Study: Project Description

The youth program, This is How We “Role,” supported by a Science Education Partnership Award (SEPA) program of the National Institute of General Medical Sciences of the National Institutes of Health, aims to diversify the veterinarian-scientist workforce by providing fun and interactive science and math experiences to K-4 students who are educationally disadvantaged due to socioeconomic status, race, or ethnicity (San Miguel et. al, 2019). Veterinarians, teachers, evaluation experts, and a children’s book illustrator developed a curriculum consisting of 56 culturally responsive, fun, and engaging youth lessons using minimal resources to expose underserved elementary school-aged students to veterinary science and careers in veterinary medicine. The curriculum was piloted and evaluated at a local community center, where veterinary medical students and veterinarians, after completing online training (i.e., training that included how to best interact and support minors, including age-appropriate/culturally responsive delivery of the curriculum), served as role models to deliver the curriculum to elementary school students. Role models were tasked with not only delivering the program, but also getting to know youth, sharing their life experiences, and showing care. The program was replicated and scaled through a sub-award system, whereby veterinary medical colleges established How We “Role” chapters with role model teams consisting of veterinarians and veterinary medical students, and a relationship with a community partner (school or community center, or other not-for-profit group) providing programming for underserved youth. Role model teams received free online training to become certified to deliver the curriculum, a starter supply package (including scissors, crayons, colored pencils, and glue sticks), curricular materials, a step-by-step guide for curriculum delivery, and assessment materials. Teams meeting IRB requirements were provided funds to support assessment costs upon receipt of assessment materials by researchers at Purdue University.

Each participating site had a How We “Role” chapter leader who was responsible for ensuring role models at their site had been certified to deliver the program and met any other institutional requirements for working with minors before they could participate. Chapter leaders notified project coordinators at Purdue University of new role models to be enrolled in the certification program and project coordinators at Purdue provided regular updated certified role model lists to chapter leaders. Remote site teams completed lesson fidelity sheets following each lesson delivered during a community partner site visit, including recording any deviations from the planned curriculum and challenges and suggested improvements for future implementations.

To implement the evaluation plan, partners attended a virtual assessment training where the evaluation team explained the IRB process and standards and introduced the approach to program evaluation, the purpose of assessment tools, and the evaluation protocol. The evaluation plan was multidimensional and included pre- and post-program surveys (including assessments of attitudes toward science, perceptions of connection to role models, and reports of demographics), pre- and post-lesson knowledge assessments, and post-lesson engagement assessments. Data collection and processing procedures were discussed in detail, and all partners were provided with an evaluation manual that detailed the evaluation procedures and included all necessary materials.

### Cross-Site Challenges

Although the How We “Role” curriculum and supply packages were all provided by the program administration, the sites were diverse in their settings and approaches. Colleges worked with different community partners at each program location. The variety of community partners introduced a bevy of contextual differences that impacted program implementation and diversity in youths served apart from the contextual differences from the implementing partners themselves (e.g., number of role models, experience interacting with and teaching youth and more). Table 1 lists potential similarities and differences across community partner sites. There are three important implications of these differences. First, there are innate differences between the types of sites: the sites could be schools, clubs or community centers. The mode of delivery varied: lessons were delivered during in-school, out-of-school, or summer camps. The frequency of delivery differed: how many lessons were taught each week across a given number of weeks. Second, participants represented different populations that may be related to the program implementation site or the youth served by the community partner (for example diversity in age, gender, and ethnicities). Third, as sites had different role models and faculty delivering the curriculum, the methods used could be systematically different across sites (time spent on group work versus relationship building). Other site geographical differences (different socioeconomic status, etc.) posed additional challenges as well. Therefore, decisions regarding how to properly combine effects of different sites when considering the aforementioned differences is complex. Hierarchical linear models are often applied to accommodate and control for site-level and youth-level differences in educational research. However, as sample sizes were small at each site, and stayed small even when pooled across sites, when compared to large-scale school studies, the performance of and interpretation of findings would be questionable.

These challenges are not unique to this specific youth outreach program. Most scaled programs delivered in more informal learning settings experience variation in implementation fidelity as serving the needs of youth and partners at a given community center is prioritized over executing rigorous research protocols. High-standards of research may also not be plausible, due to skill level of partners and available resources, or desirable, as these programs may serve young children in learning contexts where other priorities take precedence (e.g., building positive social connections, providing a safe environment, youths’ ability or desire to complete evaluation, youths’ daily attendance variations, and making sure data collections are not burdensome or exclusionary for staff or youth). However, these limitations should not impede valued programs from conducting evaluations that inform other programs and contribute to the knowledge of learning. Instead, the use of meta-analysis can enable the modeling of individual sites and the synthesis of findings across sites to help these programs make valuable research impacts.

Program coordination across sites had very few challenges. The most common challenge encountered was cancellation or rescheduling of community visits due to inclement weather or change in school schedules. In cases where program delivery deviated from plans, lesson fidelity sheets identified any differences.

### Participating Sites and Youth

In this project, 12 lessons were distributed to nine partner sites. Nine sites implemented between five and 11 lessons (*M*_lessons_ = 9). Due to incomplete or missing data, the analytic data includes eight sites and 11 lessons with a total of 340 youth. The 11 lessons covered a variety of veterinary science topics (Table 2).

Not all youth at the same site completed the same number of lessons, so sample sizes across all lessons within each site varied. For example, the “donkeys” lesson was implemented at all sites, but the “snakes” lesson reached the most youth. Table 3 displays the number of youth completing both the pre- and post-lesson knowledge assessments at each site and a breakdown of the number of lessons each site implemented.

For the analytic sample, program youth (*n* = 213) provided demographic information. They were from diverse racial and ethnic backgrounds: 32.8% White, 34.8% African American, 13.4% Hispanic, 6.0% Asian, 4.0% Native American/Pacific Islander, and 17.4% reported their ethnicity as “Other”. (Respondents could indicate more than one ethnicity/race.) There were 56.8% girls and 41.8% boys (1.4% did not respond). These students were on average 8.4 years old (Range = 5–13). The majority of students (87.3%) were between 7 and 10 years old.

### Evaluation Questions

To examine the program effects, our evaluation questions were the following:

1. Is there evidence of youth learning at each site?
2. What is the summary effect of the program on youths’ learning considering all sites together?
3. Did the differences in implementation, sample, and learning context across sites cause heterogeneous variation in learning outcomes between sites?
4. Are there any gender differences in learning outcomes of this program?

### Meta-Analysis Methods

The meta-analysis approach employs five sequential steps, all based on the calculation of site-level effect sizes. In this paper, we used Comprehensive Meta-Analysis, Version 3.3.070 to conduct the analysis.

First, standardized mean differences, or Cohen’s *d*, were calculated for each site. Cohen’s *d* is the raw difference in means divided by the standard deviation (computed within groups and pooled). Computing Cohen’s *d* for each site is an essential component of the meta-analytic approach. When Cohen’s *d* is applied in literature synthesis, this index transforms the outcomes for all studies onto a common metric. Cohen (1988) suggested that *d* = 0.2 be considered a “small” effect size, 0.5 represents a “medium” effect size and 0.8 a “large” effect size. When interpreting effect sizes, we need to convert it to a known metric. For example, if a science test’s standard deviation is 100, *d* = 0.20 means the effect is a 20-point difference.

Second, the appropriate analysis model was selected. There are two common analysis models in meta-analysis that are used when it is known that implementations differ across sites: a fixed effects model or a random effects model. In both models, we assume that effect sizes will vary across sites due to the inherent differences in context and implementation. When the purpose of an evaluation is to generalize findings to future implementations, evaluators should choose a random effects model; when the purpose of an evaluation is to produce learning about the target program and not generalize findings to future implementations, evaluators should choose a fixed effects model (Borenstein, 2019). In this multisite study, given the assumption that sites are estimating different yet related project effects, and sites with small sample sizes are important for us too, we decided in advance to pool data using a random effects meta-analysis. We chose the analysis model based on how we collected the data and our analysis purpose and made the decision before analysis.

**Figure 1.**
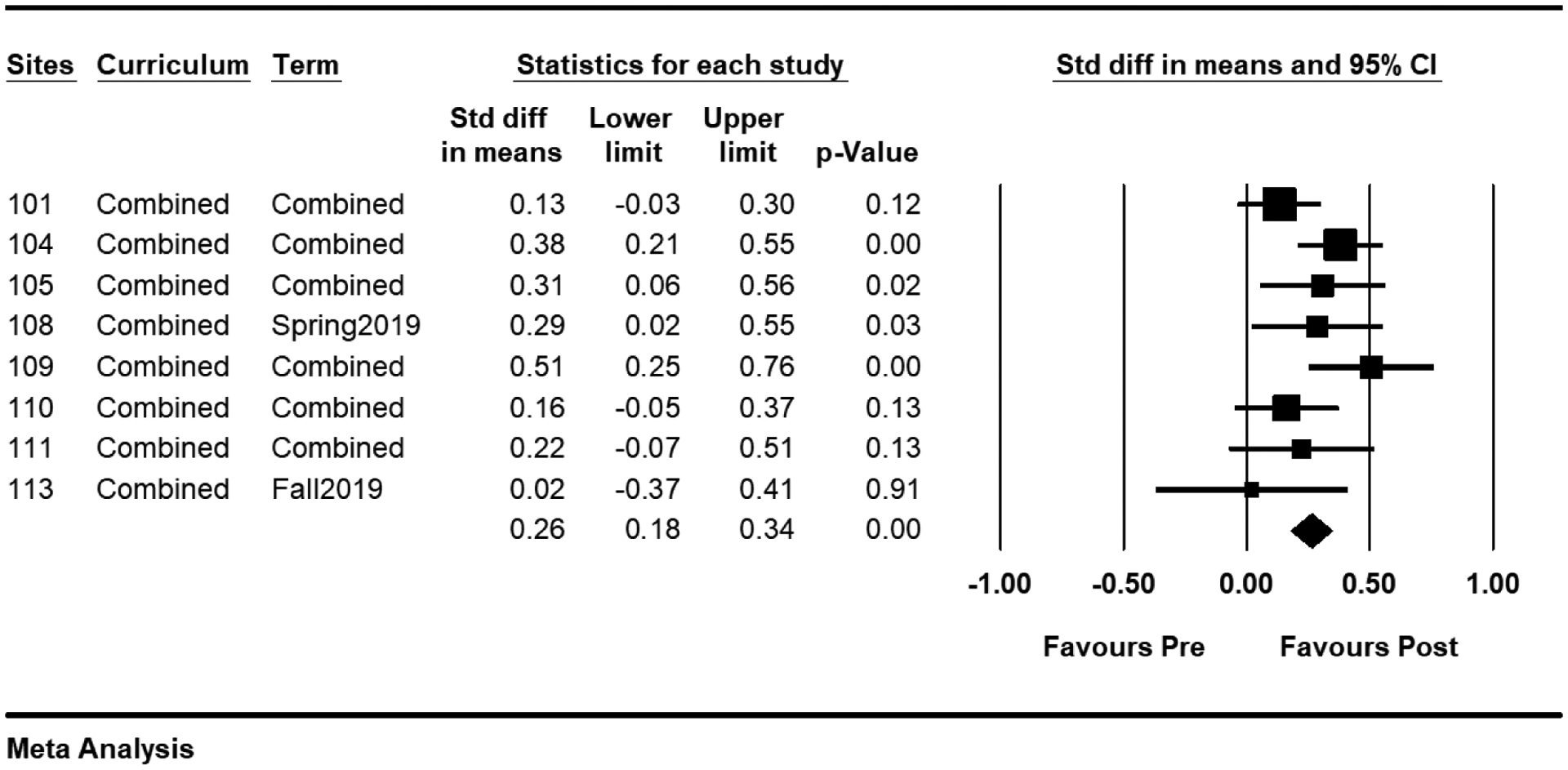
Overall Effects Forest Plot

Third, the forest plot is produced and interpreted to determine overall project impact. The forest plot is a key element in meta-analysis; it shows the effect size and precision for each site as well as the combined effect (Lalkhen, 2008). The plot has one line representing each site in the meta-analysis, plotted according to the standardized mean difference and confidence intervals (see Figure 1). It shows whether the effect sizes are consistent or varied, etc. The beginning and end of each line represents the confidence interval, and the square center on each line shows the effect size for each site. The longer the line, the wider the confidence interval, the less reliable the site results. The width of the diamond conveys the same information for the pooled results. The area of each square is proportional to the site’s weight in the meta-analysis. The bottom diamond indicates the overall effects.

Fourth, the degree of heterogeneity is estimated. The heterogeneity statistic is used to examine how much the effect sizes vary across sites. This estimate is represented by the prediction interval and it describes the distribution of true effects around the mean, whereas the confidence interval reflects the precision of the mean effect size. A prediction interval rather than a confidence interval can provide a better sense of the uncertainty around the effect estimate (Chiolero et al., 2012). When heterogeneity is innate to certain projects or programs, the various effect sizes will depend on a specific mix of populations. Effect in some populations might be harmful, even if meant to be helpful. Prediction intervals provide a range of effect sizes, within which 95% of all populations will be encompassed when considering a comparable sample. Other popular heterogeneity statistics include the chi-square statistics *Q* and *I²*. The *Q*-statistic provides a test of the null hypothesis that all sites in the analysis share a common effect size. If all sites shared the same effect sizes, the expected value of *Q* would be equal to the degrees of freedom (the number of sites minus 1). The *I²* statistics measures the proportion of heterogeneity to the total observed dispersion and is not affected by low statistical power. Higgins et al. (2003) suggested that an *I²* value of 25% might be considered low, 50% considered moderate, and 75% considered high.

Lastly, subgroups are examined. Meta-analysis allows us to compute and compare the effect size between subgroups of subjects. Subgroups analysis in meta-analysis is working with categorical factors of subjects, which is analogous to one-way ANOVA. The effect sizes calculated in the previous step are dependent variables. Relationships between subgroup and effect size never prove causality. They are observational even if all studies are random controlled trials.

## Results

We examined the potential for an overall difference across sites, using a meta-analytic approach to summarize and weight data from each of the sites (Figure 1). The left most column shows the randomly assigned site ID numbers. To estimate project overall effects, all curricula and terms were combined for each site. For site 108, the term showed “spring 2019” instead of “combined”, which means site 108 only had the program in one term spring 2019. The statistics of results for each site were displayed in the middle columns. The boxes showed the effect estimates from the single sites, while the diamond showed the pooled result. Each horizontal line through the box illustrated the length of the 95% confidence interval. The *p*-value indicates the level of statistical significance. If the diamond shape does not touch the line of no effect (0.00), the overall effect found was statistically significant, and the p-value is less than .05.

Effect sizes are an output of the analysis and are computed using means and standard deviations. For all sites, the mean effect size, the 95% confidence interval (95% CI), and the 95% prediction interval (95% PI) were computed. The forest plot showed all sites had positive mean effects (all the boxes were on the right of 0.00 vertical line). Site 109 (*d* = .51, *p* < .01 ), site 104 (*d* = .38, *p* < .01), site 105 (*d* = .31, *p* = .02), and site 108 (*d* = .29, *p* = .03) had statistically positive effects. These indicate that the How We “Role” program generally benefited the youth at each site, based on improvement of pre- and post-program knowledge assessment results, even though the program effects varied across sites.

The random-effects model was employed for the meta-analysis. The standardized mean difference was .26 (represented by the middle of diamond in the plot). On average, the project increased youths’ learning by .26 standardized deviations. The confidence interval for the standardized mean difference was .18 to .34 (the width of the diamond), which told us that the mean effect could fall anywhere in this range. This range does not include an effect size of zero, which tells us that the mean effect size is probably not zero. Similarly, the *Z*-value for testing the null hypothesis (that difference is 0.0) was 6.42, with a corresponding p-value of less than .05 (the last line of the data table). We can reject the null hypothesis and conclude that (on average) the project does increase youth learning.

We examined whether there was significant heterogeneity of effect sizes across sites (Table 4). The *Q*-value is 10.32 with 7 degrees of freedom and *p* = 0.17. We cannot reject the null hypothesis that the true effect size is identical in all the sites. The *I*² statistic is 32, which tells us that 32% of the variance in the observed effects reflects variance in true effects rather than sampling error. The variance of true effects (T²) is .01 and the standard deviation of true effects (T) is .08. The prediction interval is 0.02 to 0.50. We would expect that in some 95% of all populations comparable to those in the analysis, the true effect size will fall in this range. Based on the context outlined above, there will be some populations where the impact is trivial and some where it is medium.

**Figure 2.**
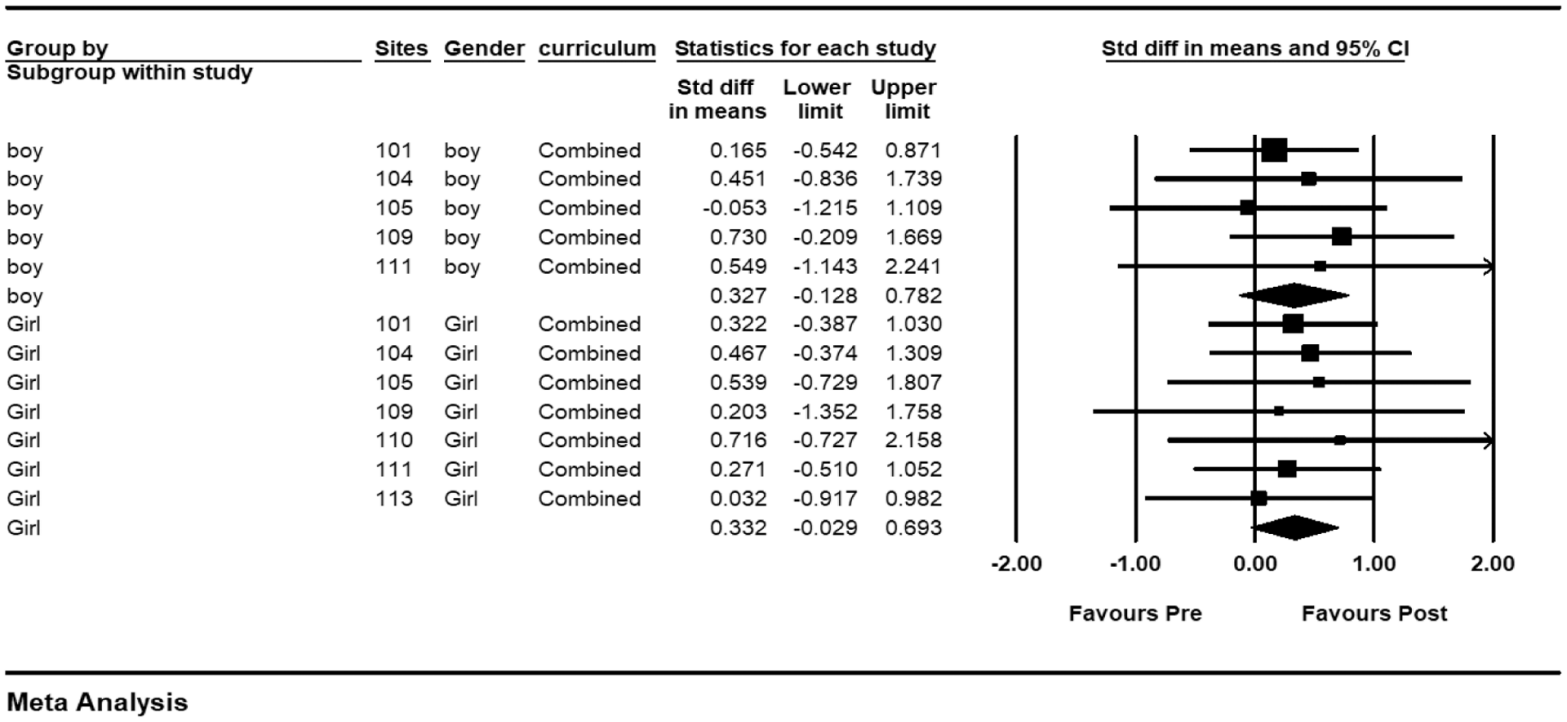
Subgroup Analysis (Gender)

As some programs may be interested in examining differences in participant demographics, we examined subgroup differences by gender even though only two thirds of the participants provided demographic information. To examine gender effects, within each site youth were classified as being girls or boys, and data were analyzed to determine if the impact of the project was greater (or smaller) for either gender. For this analysis, we used subgroups within studies as the unit of analysis. In Figure 2, the diamonds showed the subgroup mean effects for each subgroup. The mean effect for girls is .332. The mean effect size for boys is .327. The test of the difference between the two subgroups yields a *Q*-value of .0002 and *p* = .99. Thus, there is no evidence that the impact of the project varied by gender (i.e., knowledge gains were similar for girls and boys).

## Discussion

Evaluating and reporting the impact of multisite youth outreach programs is essential for continuous improvement of programs as well as garnering and maintaining program support. However, multisite studies are difficult to conduct in highly controlled ways and often produce data from heterogeneous contexts that make data analysis and appropriate interpretations difficult. The variability across sites increases generalizability and external validity but complicates data analysis and interpretations. We used the evaluation of the nationwide implementation of This Is How We “Role” as an example to illustrate how to use meta-analysis to analyze data of multisite youth outreach programs. We computed site-specific effect sizes, then calculated overall mean effect sizes, and used a forest map to display the effect size and precision for each study as well as the combined effect. We also assessed heterogeneity of effect sizes among sites and gave an example of subgroup analysis.

Results showed the How We “Role” program generally increased learning at each site, even though the program effects varied across sites. Overall, the project increased youth learning by .26 standardized deviations. For 95% of all populations comparable to those in the analysis, the true effect size will fall between 0.02 and 5.0. Based on the context outlined above, there will be some populations where the impact is trivial and some where it is medium. No subgroup differences were observed between girls and boys. This data was used to inform further curricular development by including activities and delivery methods that resulted in greatest impact on learning. Synthesizing the individual effect of each sites into a single pooled effect characterized by statistical precision gave us a greater confidence that the How We “Role” program enhanced youth learning of veterinary science.

The How We “Role” Program evaluation included other assessment tools including participants’ pre- and post-program perceptions of social connection with role models and attitudes toward science, and post-lesson perceptions of engagement. However, many youth did not complete both the pre-and post-program assessments. Therefore, we were unable to examine change in these variables over the course of the program and, as a result, this data was not included in the current analysis. Due to inconsistencies of data collection, the sample for each instrument analysis varied and represented different sub-samples of all youth who participated in the program.

Meta-analysis has become a well-known method for synthesis of quantitative data (Bax et al., 2006). Meta-analysis is useful for evaluating multisite youth outreach programs, especially during scaling, when sites are not homogeneous, have limited sample sizes, and no control group. In addition, the effect sizes are metric free and very flexible. They can be compared across outcomes or indicators as well as across other relevant variables. Thus, meta-analysis can accommodate a wide variety of evaluation designs, and can be applied to estimate gain scores, correlation coefficients, odds ratios, risk ratios, point estimates, and more. Additionally, meta-analysis provides details of the underlying variation between and within sites, while most traditional multisite evaluation methods cannot. Advantages of using meta-analysis in multisite programs also include seeing the “landscape” of the multisite program, keeping statistical significance in perspective, minimizing wasted data, becoming intimate with the data summarized, asking focused research questions, and finding moderator variables (Rosenthal & DiMatteo, 2001). An evident weakness of multisite meta-analysis is that it will not capture qualitative distinctions between sites, and when examining data from each site, it requires a good deal of effort to understand resultant differences and/or similarities.

A meta-analysis is a synthesis. How we approach the synthesis depends on our goals. When we use meta-analysis in evaluating multisite programs, it is not selecting studies from the literature in a field, and there is no need to estimate publication bias, which are two major challenges in literature synthesis meta-analysis. In the application of multisite meta-analysis, however, we should be sure to select an analysis model based on our data and purpose and we should not change models after seeing the heterogeneity results. Further, we should avoid another common mistake and use the prediction interval to examine heterogeneity across sites. *I*² can tell us only that the percentage of the variance in the observed effects reflects variance in true effects rather than sampling error. However, the prediction interval indicates how the true effect size varies across populations, and it does so on a scale that allows us to address the utility of the intervention for all sites, no matter their degree of disconformity (Borenstein et al., 2019).

This work intends to benefit evaluators working with multisite youth outreach programs, especially uncontrolled studies with heterogeneous data. This paper reports on the multisite meta-analysis methods: how to choose an appropriate statistical model for multi-site youth programs, the differences between multisite meta-analysis and traditional literature synthesis meta-analysis, how to address heterogeneity, how to interpret effect sizes and findings, and other statistical roadblocks. We discussed the process of multisite meta-analysis as well as on the challenges associated with this work, such as selecting models, effect size and confidence interval interpretation, subgroup analysis, examining heterogeneity, common mistakes and questions, etc. This introduction and application of this approach will be of interest to evaluators who are planning or implementing multisite evaluations and want to best capture the potential effects of their program using a robust and appropriate statistical approach. Indeed, meta-analysis neither requires significant compromises of the program integrity to maintain analysis rigor or the compromise of analysis rigor to maintain program integrity. Thus, meta-analysis enables evaluators to better understand and capture the important effects of this program and prioritizes the values and purpose of youth outreach programs.

## Figures and Tables

**Table 1.** Similarities and Differences Across Sites

Similarities across sites	Differences across sites
The partnership application process was the same.	Community partners could be schools, clubs or community centers.
Teams consisted of a college and community partners with a minimum of 6 role models from each college. A faculty/staff member in the college served as the PI.	Universities recruited veterinary medical students using their own criteria. (These students needed to be certified as role models before they could teach the youth.)
Role models were all veterinary medical students. All role models experienced the same certification process (including teaching methods training).	Role models were different in demographics (ethnicity, background, etc.).
At least one role model helped deliver each lesson.	Sites had different student-to-role model ratios (some sites might have more role models).
The curricular structure supplied for each topic was the same.	Role models could modify curricula to leverage their personal strengths, expertise, and the needs of the youth they serve. Sites might have different frequency of visits to the community partners.
All sites received the same first batch of curricula. Sites that requested a second batch of curricula all received the same second batch.	Sites completed different numbers of lessons. (Some sites did not request the second batch of curricula.)
	Instruction happened in different settings: during school days, after school, or at summer camps.
	Different youth demographics (age, race, ethnicity, socioeconomic status etc.)

**Table 2.** Science Topics in Each Lesson

Lesson title	Lesson topic
Careers in Veterinary Medicine	Youth learned about the career opportunities in the veterinary profession and some of the tools that veterinarians use.
Donkeys Need Dentists, Too!	Youth learned about teeth. Dental examinations for people and other animals were compared.
Elephants Need Eye Doctors, Too!	Youth learned about eyes. Eye examinations for people and other animals were compared.
Fish Veterinarians	Youth learned about the role of fish veterinarians and what fish need to stay healthy.
Government Veterinarians	Youth learned about jobs that government veterinarians perform, the importance of animal identification, and how veterinarians determine where a disease originated and where it might have spread.
Let’s Do Research!	Youth learned about research and why research is important. Youth defined and developed hypotheses, learned about experimental design, practiced data collection through scientific observation and recording information, analyzed data, and drew conclusions.
Muscles	Youth learned the major muscles of people and animals, explored how muscles contract and expand, and compared similarities and differences between muscles.
Poultry Veterinarians	Youth learned about the role of poultry veterinarians and what poultry need to stay healthy.
Puppy and Kitten Math	Youth practiced math skills in the context of costs associated with owning a dog or cat.
Snakes, Turtles, and Tortoises	Youth learned how veterinarians provide healthcare for reptiles and what snakes, turtles, and tortoises need to stay healthy.
Take a Look Inside	Youth learned the anatomy and function of the major body organs. Youth compared organs of rats, dogs, cats, pigs, cows, horses, goats, and people.

**Table 3.** Youth and Lessons in Each Site

Site (no. of lessons)	Careers in Vet Med	Donkeys Need Dentists, Too!	Elephants Need Eye Doctors, Too!	Fish Vets	Government Vets	Let’s Do research!	Muscles	Poultry Vets	Puppy and Kitten Math	Snakes, Turtles, and Tortoises	Take a Look Inside	*N*
101 (8)		✕	✕		✕	✕	✕	✕		✕	✕	101
104 (10)	✕	✕	✕	✕	✕	✕	✕		✕	✕	✕	48
105 (10)		✕	✕	✕	✕	✕	✕	✕	✕	✕	✕	38
108 (6)	✕	✕		✕				✕	✕	✕		28
109 (10)		✕	✕	✕	✕	✕	✕	✕	✕	✕	✕	40
110 (9)		✕		✕	✕	✕	✕	✕	✕	✕	✕	25
111 (4)		✕				✕	✕			✕		49
113 (5)	✕	✕		✕				✕	✕			11
												340

*Note*. *N* = Number of youth who took the pre- and post-program assessments.

**Table 4.** Heterogeneity Statistics

Model	*Q*-value	*df* (Q)	*p*-value	*I* squared	Tau Squared	Standard Error	Variance	Tau
Random	10.32	7	.17	32.17	0.01	0.01	0.00	0.08
